# Mobile Health for All: Public-Private Partnerships Can Create a New Mental Health Landscape

**DOI:** 10.2196/mental.5843

**Published:** 2016-06-06

**Authors:** Dror Ben-Zeev

**Affiliations:** ^1^ Dartmouth College Lebanon, NH United States

**Keywords:** mHealth, policy, reimbursement, access

## Abstract

Research has already demonstrated that different mHealth approaches are feasible, acceptable, and clinically promising for people with mental health problems. With a robust evidence base just over the horizon, now is the time for policy makers, researchers, and the private sector to partner in preparation for the near future. The Lifeline Assistance Program is a useful model to draw from. Created in 1985 by the U.S. Federal Communications Commission (FCC), Lifeline is a nationwide program designed to help eligible low-income individuals obtain home phone and landline services so they can pursue employment, reach help in case of emergency, and access social services and healthcare. In 2005, recognizing the broad shift towards mobile technology and mobile-cellular infrastructure, the FCC expanded the program to include mobile phones and data plans. The FCC provides a base level of federal support, but individual states are responsible for regional implementation, including engagement of commercial mobile phone carriers. Given the high rates of disability and poverty among people with severe mental illness, many are eligible to benefit from Lifeline and research has shown that a large proportion does in fact use this program to obtain a mobile phone and data plan. In the singular area of mobile phone use, the gap between people with severe mental illness and the general population in the U.S. is vanishing. Strategic multi-partner programs will be able to grant access to mHealth for mental health programs to those who will not be able to afford them—arguably, the people who need them the most. Mobile technology manufacturing costs are dropping. Soon all mobile phones in the marketplace, including the more inexpensive devices that are made available through subsidy programs, will have “smart” capabilities (ie, internet connectivity and the capacity to host apps). Programs like Lifeline could be expanded to include mHealth resources that capitalize on “smart” functions, such as secure/encrypted clinical texting programs and mental health monitoring and illness-management apps. Mobile phone hardware and software development companies could be engaged to add mHealth programs as a standard component in the suite of tools that come installed on their mobile phones; thus, in addition to navigation apps, media players, and games, the new Android or iPhone could come with guided relaxation videos, medication reminder systems, and evidence-based self-monitoring and self-management tools. Telecommunication companies could be encouraged to offer mHealth options with their data plans. Operating system updates pushed out by the mobile carrier companies could come with optional mHealth applications for those who elect to download them. In the same manner in which the Lifeline Assistance Program has helped increase access to fundamental opportunities to so many low-income individuals, innovative multi-partner programs have the potential to put mHealth for mental health resources in the hands of millions in the years ahead.

We are closer than ever to having viable mobile health (mHealth) options for people with psychiatric illnesses and must ensure that the most vulnerable members of society will be able to benefit from the opportunities these clinical technologies can provide. Worldwide, the majority of the adult population has access to mobile devices [1]. Even people with severe mental illnesses, who have historically been the last to gain access to technological innovations, now own and use mobile phones [2]. Like the general population, people with psychiatric conditions are interested in leveraging their personal mobile devices to enhance their health, as demonstrated in numerous publications in this and other journals [3-5]. Clinical researchers across continents have responded to the public need by developing innovative mHealth approaches that use a range of mobile device functions such as texting, apps, and sensors for clinical assessment and treatment. The US National Institute of Mental Health, the largest funder of mental health research in the world, has already supported hundreds of technology-based studies, and mHealth for mental health projects continue to be funded annually. Private sector technology companies such as Google, Apple, and IBM as well as pharmaceutical companies are moving into the mHealth arena with an eye toward mental health. Research has already demonstrated that different mHealth programs are feasible, acceptable, and clinically promising for people with mental health problems [6-9]. A robust evidence base supporting the utility of different mHealth approaches is just over the horizon. Now is the time for policy makers, researchers, and the private sector to partner in preparation for the near future.

The Lifeline Assistance Program is a useful model to draw from [10]. Created in 1985 by the US Federal Communications Commission (FCC), Lifeline is a nationwide program designed to help eligible low-income individuals obtain home phone and landline services so they can pursue employment, reach help in case of emergency, and access social services and health care. In 2005, recognizing the broad shift toward mobile technology and mobile-cellular infrastructure, the FCC expanded the program to include mobile phones and data plans. The FCC provides a base level of federal support, but individual states are responsible for regional implementation, including engagement of commercial mobile phone carriers. Given the high rates of disability and poverty among people with severe mental illness, many are eligible to benefit from Lifeline and research has shown that a large proportion does in fact use this program to obtain a mobile phone and data plan [2]. In the singular area of mobile phone use, the gap between people with severe mental illness and the general population in the United States is vanishing.

Strategic multi-partner programs will be able to grant access to mHealth for mental health programs to those who will not be able to afford them—arguably, the people who need them the most. Mobile technology manufacturing costs are dropping. Soon all mobile phones in the marketplace, including the more inexpensive devices that are made available through subsidy programs, will have “smart” capabilities (ie, Internet connectivity and the capacity to host apps). Programs like Lifeline could be expanded to include mHealth resources that capitalize on “smart” functions, such as secure/encrypted clinical texting programs and mental health monitoring and illness-management apps. Mobile phone hardware and software development companies could be engaged to add mHealth programs as a standard component in the suite of tools that come installed on their mobile phones; thus, in addition to navigation apps, media players, and games, the new Android or iPhone could come with guided relaxation videos, medication reminder systems, and evidence-based self-monitoring and self-management tools. Telecommunication companies could be encouraged to offer mHealth options with their data plans. Operating system updates pushed out by the mobile carrier companies could come with optional mHealth apps for those who elect to download them. In the same manner in which the Lifeline Assistance Program has helped increase access to fundamental opportunities to so many low-income individuals, innovative multi-partner programs have the potential to put mHealth for mental health resources in the hands of millions in the years ahead.

*Dror Ben-Zeev, PhD*

**Figure 1:**
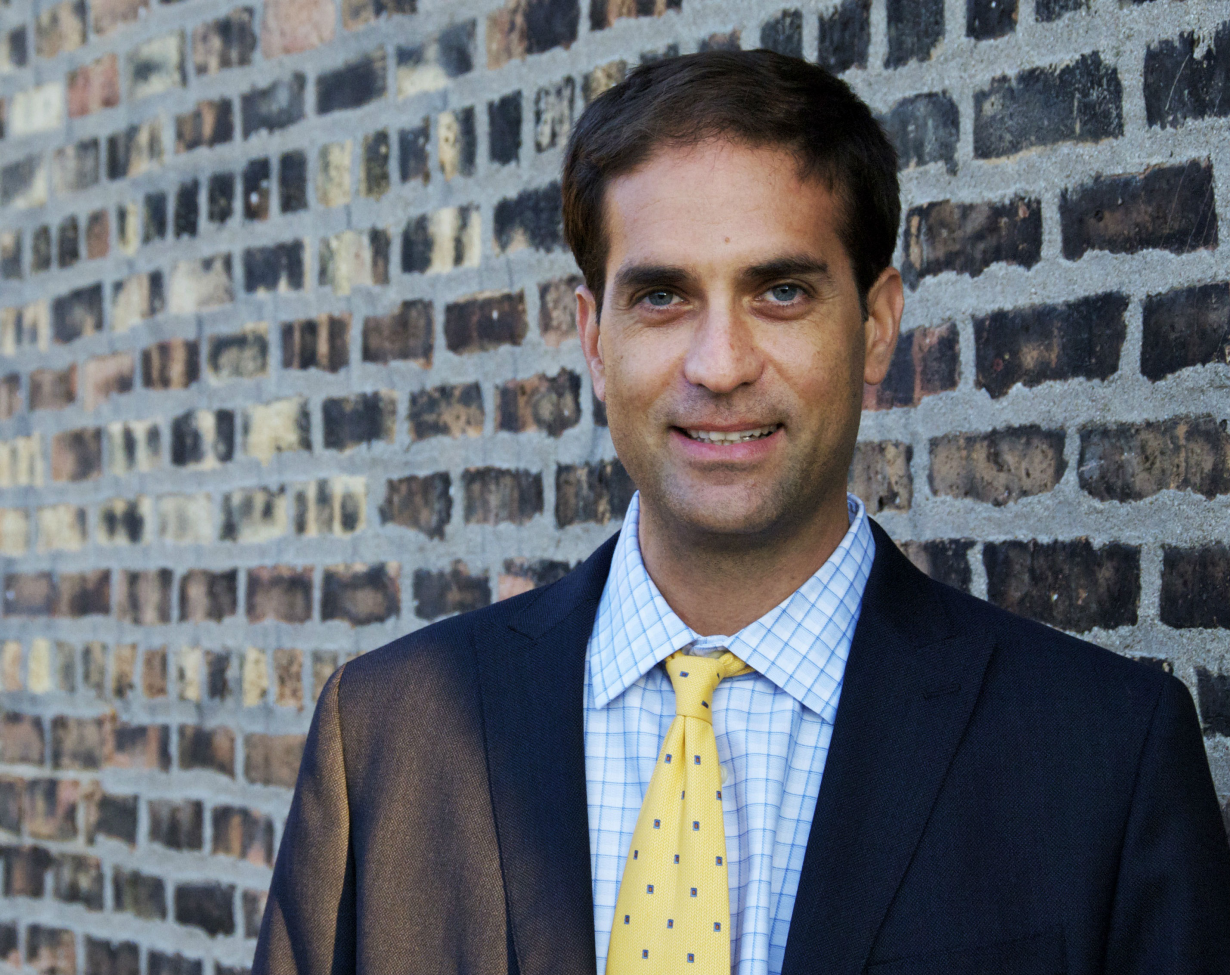
Dror Ben-Zeev, PhD, is a faculty member in the Department of Psychiatry at Dartmouth College and licensed Clinical Psychologist who specializes in development and evaluation of technology-based approaches in the study, assessment, and treatment of mental illness. Dr. Ben-Zeev serves as the Director of the Mobile Health (mHealth) for Mental Health Program, a multidisciplinary effort to harness mobile technology (e.g., texting, smartphone applications, multi-modal sensors) to improve the outcomes and support the recovery of people with psychiatric conditions.

## Abbreviations

FCC: US Federal Communications Commission
mHealth: mobile health

## References

[1] International Telecommunication Union (2015). The world in 2015: ICT facts and figures.

[2] Ben-Zeev D, Davis KE, Kaiser S, Krzsos I, Drake RE (2013). Mobile technologies among people with serious mental illness: opportunities for future services. Adm Policy Ment Health.

[3] Firth J, Cotter J, Torous J, Bucci S, Firth J, Yung A (2015). Mobile Phone Ownership and Endorsement of ?mHealth? Among People With Psychosis: A Meta-analysis of Cross-sectional Studies. Schizophr Bull.

[4] Proudfoot J, Parker G, Hadzi PD, Manicavasagar V, Adler E, Whitton A (2010). Community attitudes to the appropriation of mobile phones for monitoring and managing depression, anxiety, and stress. J Med Internet Res.

[5] Torous J, Chan SR, Tan SY, Behrens J, Mathew I, Conrad EJ, Hinton L, Yellowlees P, Keshavan M (2014). Patient smartphone ownership and interest in mobile apps to monitor symptoms of mental health conditions: A survey in four geographically distinct psychiatric clinics. JMIR Mental Health.

[6] Ainsworth John, Palmier-Claus Jasper E, Machin Matthew, Barrowclough Christine, Dunn Graham, Rogers Anne, Buchan Iain, Barkus Emma, Kapur Shitij, Wykes Til, Hopkins Richard S, Lewis Shôn (2013). A comparison of two delivery modalities of a mobile phone-based assessment for serious mental illness: native smartphone application vs text-messaging only implementations. J Med Internet Res.

[7] Ben-Zeev D, Brenner CJ, Begale M, Duffecy J, Mohr DC, Mueser KT (2014). Feasibility, acceptability, and preliminary efficacy of a smartphone intervention for schizophrenia. Schizophr Bull.

[8] Faurholt-Jepsen M, Frost M, Vinberg M, Christensen EM, Bardram JE, Kessing LV (2014). Smartphone data as objective measures of bipolar disorder symptoms. Psychiatry Res.

[9] Kannisto K, Adams C, Koivunen M, Katajisto J, Välimäki M (2015). Feedback on SMS reminders to encourage adherence among patients taking antipsychotic medication: a cross-sectional survey nested within a randomised trial. BMJ Open.

[10] FCC Lifeline Assistance Program.

